# Genome-wide association study of cooking-caused grain expansion in rice (*Oryza sativa* L.)

**DOI:** 10.3389/fpls.2023.1250854

**Published:** 2023-08-30

**Authors:** Yan Zheng, Khin Mar Thi, Lihui Lin, Xiaofang Xie, Ei Ei Khine, Ei Ei Nyein, Min Htay Wai Lin, Win Win New, San San Aye, Weiren Wu

**Affiliations:** ^1^ College of Life Sciences, Fujian Agriculture and Forestry University, Fuzhou, Fujian, China; ^2^ Fujian Provincial Key Laboratory of Crop Breeding by Design, Fujian Agriculture and Forestry University, Fuzhou, Fujian, China; ^3^ Key Laboratory of Genetics, Breeding and Multiple Utilization of Crops, Ministry of Education, Fujian Agriculture and Forestry University, Fuzhou, Fujian, China; ^4^ Department of Botany, Mawlamyine University, Mawlamyine, Myanmar

**Keywords:** rice, grain breadth expansion index (GBEI), grain length expansion index (GLEI), grain length-breadth relative expansion index (GREI), Genome-wide association study (GWAS)

## Abstract

Cooking-caused rice grain expansion (CCRGE) is a critical trait for evaluating the cooking quality of rice. Previous quantitative trait locus (QTL) mapping studies on CCRGE have been limited to bi-parental populations, which restrict the exploration of natural variation and mapping resolution. To comprehensively and precisely dissect the genetic basis of CCRGE, we performed a genome-wide association study (GWAS) on three related indices: grain breadth expansion index (GBEI), grain length expansion index (GLEI), and grain length-breadth ratio expansion index (GREI), using 345 rice accessions grown in two years (environments) and 193,582 SNP markers. By analyzing each environment separately using seven different methods (3VmrMLM, mrMLM, FASTmrMLM, FASTmrEMMA, pLARmEB, pKWmEB, ISIS EM-BLASSO), we identified a total of 32, 19 and 27 reliable quantitative trait nucleotides (QTNs) associated with GBEI, GLEI and GREI, respectively. Furthermore, by jointly analyzing the two environments using 3VmrMLM, we discovered 19, 22 and 25 QTNs, as well as 9, 5 and 7 QTN-by-environment interaction (QEIs) associated with GBEI, GLEI and GREI, respectively. Notably, 12, 9 and 15 QTNs for GBEI, GLEI and GREI were found within the intervals of previously reported QTLs. In the vicinity of these QTNs or QEIs, based on analyses of mutation type, gene ontology classification, haplotype, and expression pattern, we identified five candidate genes that are related to starch synthesis and endosperm development. The five candidate genes, namely, *LOC_Os04g53310* (*OsSSIIIb*, near QTN *qGREI-4.5s*), *LOC_Os05g02070* (*OsMT2b*, near QTN *qGLEI-5.1s*), *LOC_Os06g04200* (*wx*, near QEI *qGBEI-6.1i* and QTNs *qGREI-6.1s* and *qGLEI-6.1t*), *LOC_Os06g12450* (*OsSSIIa*, near QTN *qGLEI-6.2t*), and *LOC_Os08g09230* (*OsSSIIIa*, near QTN *qGBEI-8.1t*), are predicted to be involved in the process of rice grain starch synthesis and to influence grain expansion after cooking. Our findings provide valuable insights and will facilitate genetic research and improvement of CCRGE.

## 1 Introduction

Rice (*Oryza sativa* L.) is a crucial cereal crop that serves as a staple food for over half of the global population. It is the only cereal crop that is primarily consumed as whole grains, which underscores its significance in the field of rice breeding (Hossain et al., 2009). The quality of rice is assessed based on several factors, including appearance, milling, cooking, sensory properties, and nutrition (Cheng et al., 2005; Feng et al., 2017). Among these factors, cooking quality is a critical determinant for the economic value of rice. The cooking quality of rice refers to the characteristics of cooked rice, including its texture, tenderness, stickiness, and overall palatability. As starch accounts for up to 95% of the dry weight of a polished rice grain (Fitzgerald et al., 2009), the cooking quality of rice is mainly determined by starch. During the cooking process, rice grains absorb water and undergo gelatinization, leading to a noticeable expansion in volume (Golam and Prodhan, 2013). The extent of this cooking-caused rice grain expansion (CCRGE) can affect the texture, tenderness and overall quality of cooked rice, and is significantly influenced by the properties of starch (Pang et al., 2016). In general, rice varieties with a higher amylose content (AC) tend to absorb more water and exhibit greater increase in volume after cooking (Frei et al., 2003). Hence, CCRGE is a complex trait closely related to the cooking quality of rice. As the desired cooking quality can vary depending on the type of rice and the culinary preferences of individuals or cultural cuisines (Suwannaporn and Linnemann, 2008), the corresponding suitable degree of CCRGE is also diverse. To meet the varying demands for the cooking quality of rice, different goals should be established in rice breeding. Dissecting the genetic basis of CCRGE will facilitate the efforts toward the goals.

For this purpose, a number of studies have been conducted to map quantitative trait loci (QTLs) underlying CCRGE. To date, 47 QTLs for grain length expansion (Ahn et al., 1993; Li et al., 2004; Zhang et al., 2004; Ge et al., 2005; Shen et al., 2005; Tian et al., 2005; Wang et al., 2007; Amarawathi et al., 2008; Liu et al., 2008; Govindaraj et al., 2009; Shen et al., 2011; Swamy et al., 2012; Li et al., 2015; Arikit et al., 2019), 10 QTLs for grain breadth expansion (Ge et al., 2005; Govindaraj et al., 2009), and 15 QTLs for grain length-breadth relative expansion (He et al., 2003; Jiang et al., 2008; Liu et al., 2008; Thi et al., 2020; Malik et al., 2022) have been reported, demonstrating that CCRGE is a very complex trait. However, none of these QTLs have been cloned.

All the QTLs reported for CCRGE were identified through conventional linkage analysis methods utilizing various populations derived from bi-parental crosses, including F_2_ (Arikit et al., 2019), F_3_ (Ahn et al., 1993), F_2:3_ (Jiang et al., 2008; Thi et al., 2020), BC_2_F_2_ (Swamy et al., 2012), BC_3_F_1_ (Li et al., 2004), doubled haploid (DH) (Zhang et al., 2004; Tian et al., 2005; Govindaraj et al., 2009), and recombinant inbred lines (RILs) (He et al., 2003; Malik et al., 2022). The linkage-based QTL mapping methods are limited by two main factors. First, it can only investigate the variation between two parents. Second, it has a low mapping resolution due to strong linkage disequilibrium in the mapping population used. Consequently, the mapped QTLs can only account for a small portion of the related genetic variations in the rice germplasm. Therefore, further studies are necessary.

During the domestication process, rice germplasm resources have accumulated a rich array of natural variations in the genome. The advent of high-throughput DNA sequencing technologies has facilitated the use of genome-wide association study (GWAS) as an effective method for identifying natural genomic variations associated with quantitative traits (Huang et al., 2010; Zhao et al., 2011). Unlike the linkage-based QTL mapping method, GWAS utilizes high-density single nucleotide polymorphisms (SNPs) as genetic markers and is performed on diverse natural populations. As linkage disequilibrium is much weaker in natural populations, GWAS achieves higher resolution in QTL mapping (Huang and Han, 2014; Burghardt et al., 2017). GWAS has been successfully employed to map genes or QTLs for numerous important traits in rice, such as flowering time (Huang et al., 2012), grain yield components (Eizenga et al., 2019), grain qualities (Misra et al., 2017; Wang et al., 2020), and so on. However, to date, no GWAS has been conducted to identify QTLs underlying CCRGE.

In this study, we performed GWAS on three traits of CCRGE based on two replicated experiments conducted in two different years (environments) and using seven different methods to analyze the data. We detected 165 related quantitative trait nucleotides (QTNs), including some exhibiting only the effect of QTN-by-environment interaction (QEI). Based on the detected QTNs, we identified five candidate genes through gene ontology (GO), haplotype, and expression pattern analyses. Our findings will facilitate further genetic research and the genetic improvement of CCRGE.

## 2 Materials and methods

### 2.1 Plant materials and field experiments

A set of 345 rice accessions among the list of the 3K Rice Genomes Project (2014) were utilized for this research (**Table S1**). These accessions included 108 japonica, 177 indica, 48 circum-Aus group (cA), 2 circum-Basmati group (cB), and 10 admixed (between major groups) according to Wang et al. (2018). All accessions were grown at the Experimental Farm of Fujian Agriculture and Forestry University in Yangzhong (E118.485841, N26.287161) during the normal growing season (April to October) in 2017 (E1) and 2018 (E2). In both years, 20 seeds of each accession were sown on a seedbed after pregermination, and 14 seedlings were transplanted onto the paddy field 25 days later with a 20-cm spacing between plants and between rows. Field management followed standard agronomic procedures. Mature seeds were harvested from each accession, and subjected to sun, then stored at the room temperature. The newly harvested seeds were utilized for the measurement of CCRGE traits in each year.

### 2.2 Measure of cooking-caused grain expansion

The procedure for quantifying the characteristics of cooking-caused rice grain expansion was performed according to Thi et al. (2020). The experiment was conducted in three replicates for each accession. In each replicate, 30 intact white rice grains were soaked (for 30 min) and boiled (for 45 min), and the average length and average breadth of 30 uncooked grains (L_0_ and B_0_) and 15 unbroken and straight cooked grains (L_1_ and B_1_) were measured. Subsequently, the grain breadth expansion index (GBEI), grain length expansion index (GLEI) and grain length-breadth relative expansion index (GREI) of each accession were calculated according to the formulae described by Thi et al. (2020), where GLEI = L_1_/L_0_, GBEI = B_1_/B_0_, and GREI = (L_1_/B_1_)/(L_0_/B_0_) = (L_1_/L_0_)/(B_1_/B_0_) = GLEI/GBEI.

### 2.3 Collection of SNP data

The SNP data of the 345 rice accessions were obtained from the 3K Rice Genomes Project (http://iric.irri.org/resources/3000-genomes-project). The core genome set of 404K SNPs (https://snp-seek.irri.org/download.zul, accessed on 1 September 2021) was employed for the analysis. A stringent quality control process was performed, which involved removal of the SNPs that had more than 20% missing calls and a minor allele frequency (MAF) smaller than 5%. As a result, a total of 193,582 SNPs were retained for subsequent analysis.

### 2.4 Clustering, population structure and linkage disequilibrium analyses

The genetic distances between 345 accessions were calculated based on SNP data, and a phylogenetic tree was constructed using the MEGA 11 software. Population structure was analyzed using principal component analysis (PCA) plots and the Admixture program as described by Alexander and Lange (2011). The linkage disequilibrium (LD) between pairwise SNPs located within 1 megabase (Mb) on each chromosome or across the entire genome was estimated by computing the determination coefficient (R^2^) using the plink software (Purcell et al., 2007).

### 2.5 Genome-wide association studies

GWAS was performed on GLEI, GBEI and GREI with two strategies: (1) single-environment analysis, namely, analyzing each environment separately; and (2) two-environment analysis, namely, analyzing the two environments jointly. For single-environment analysis, we employed two R packages: 3VmrMLM (Li et al., 2022; https://github.com/YuanmingZhang65/IIIVmrMLM) and mrMLM v4.0.2 (Zhang et al., 2020). The former includes the method 3VmrMLM, while the latter contains six methods, namely, mrMLM (Wang et al., 2016), FASTmrMLM (Tamba and Zhang, 2018), FASTmrEMMA (Wen et al., 2018), pLARmEB (Zhang et al., 2017), pKWmEB (Ren et al., 2018), and ISIS EM-BLASSO (Tamba et al., 2017). The option “method=Single_env” was chosen in 3VmrMLM, while default parameters were used for the other methods. Two-environment analysis was conducted using 3VmrMLM only, with the option set to “method=Multi_env”. This method allowed for the estimation of the main effect of a QTN and the effect of QTN-by-environment interaction. For distinction, a QTN showing only the effect of QTN-by-environment interaction was denoted as QEI. Each QTN or QEI was named following the nomenclature “q + trait + chromosome + number + s/t/i”, where “s” and “t” indicate that the QTN was detected based on single- or two-environment analysis, respectively, and “i” indicates a QEI. According to Zhang et al. (2019), the QTNs identified by multiple methods were deemed as reliable QTNs, with particular emphasis on those identified in multiple environments, which were considered stable QTNs.

### 2.6 Prediction of candidate genes

Based on the distinct LD decay in each rice chromosome, the left and right R^2^ half-decay regions flanking each QTN or QEI were determined to identify potential candidate genes. The following sequential steps were executed: (1) the SNP effect prediction software snpEff.v1.9 (Cingolani et al., 2012) was employed to evaluate the effects of SNPs on the regional genes, and annotated genes with effective mutation types, such as non-synonymous substitution, splice site, and UTR-5’ mutation, were selected; (2) GO classifications related to starch synthesis or endosperm development were searched in the rice database (https://www.ricedata.cn/ontology/), and all genes with these GO classifications were retrieved; and (3) genes that meet both steps 1 and 2 were screened out and then subjected to haplotype analysis, where different haplotypes exhibiting *t*-test significance were considered as candidate genes.

### 2.7 Tissue specific expression of candidate genes

The expression profiles of the candidate genes in various tissues were obtained from the Rice Genome Annotation Project database (http://rice.uga.edu), including shoots (library name in NCBI: SRR042529), leaves-20 days (OSN_AA and OSN_CA), pre-emergence inflorescence (OSN_AC), post-emergence inflorescence (OSN_AB), anther (OSN_AD), pistil (OSN_AE), seed-5 DAP (days after pollination; OSN_AF), seed-10 DAP (OSN_AK), embryo-25 DAP (OSN_AG) and endosperm-25 DAP (OSN_AH and OSN_BH). A heatmap was generated to visualize the gene expression patterns across the different tissues.

## 3 Results

### 3.1 Trait performance

**Figure 1:**
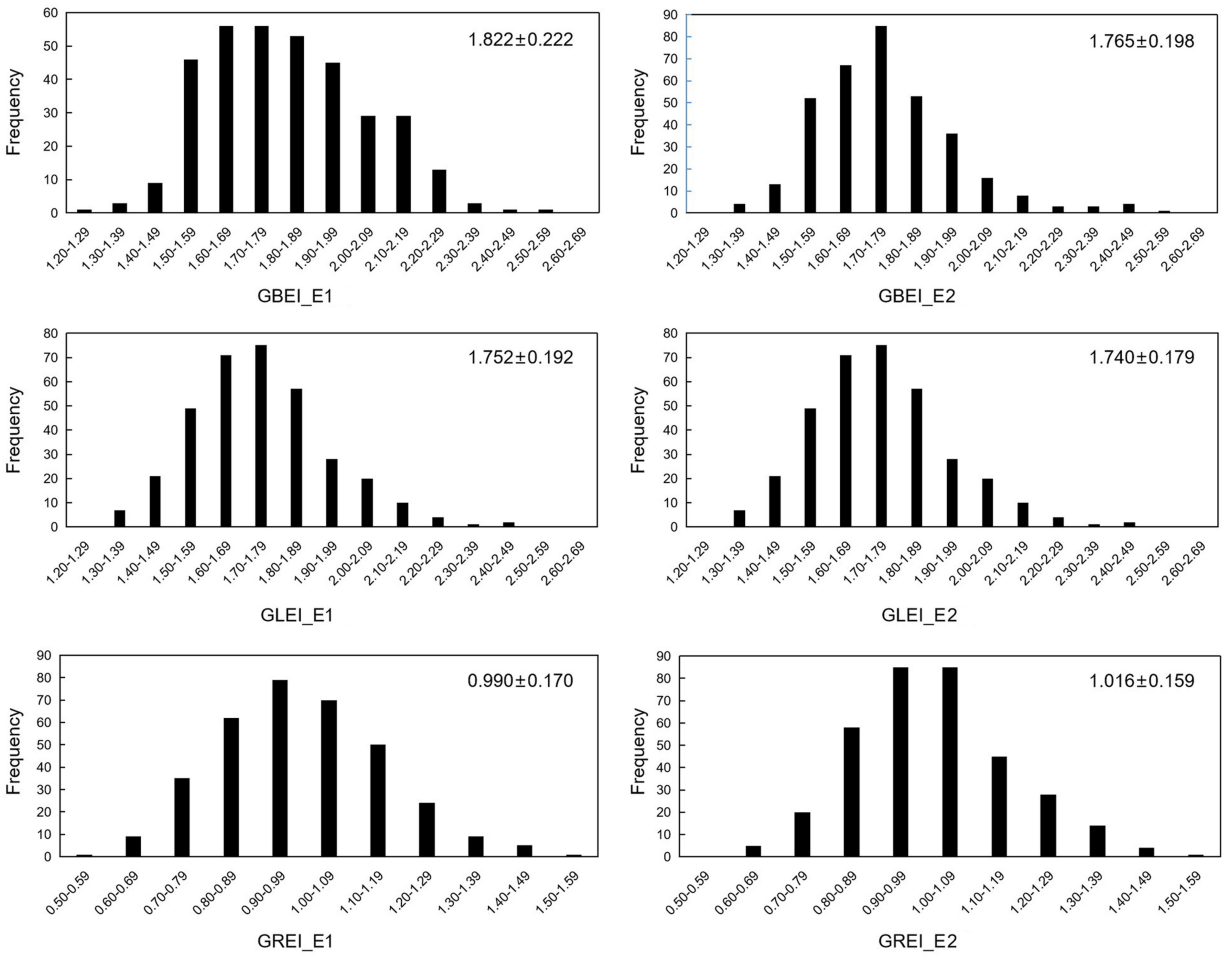
Frequency distribution of GBEI, GLEI and GREI in two environments. Values on the top right corner of each diagram are mean ± standard deviation (cm).

The traits GBEI, GLEI, and GREI exhibited a continuous unimodal distribution in both environments, suggesting that these traits are quantitative and controlled by multiple genes (**Figure 1**). After performing the Brown-Forsythe Test for assessing homogeneity of variances, the analysis revealed that the error variances of each accession in both environment for the three traits were homogeneous, indicating that the collected data is suitable for subsequent analysis of variance (ANOVA). Although the population means of these traits were similar in both environments (GBEI: 1.822 and 1.765; GLEI: 1.752 and 1.740; GREI: 0.990 and 1.016), ANOVA revealed statistically significant variation between the two environments and genotype-by-environment interaction (**Table 1**). These results indicated that all the three traits exhibited significant variation across macro-environments. However, there were still significant correlations between the two environments in these traits, particularly in GLEI and GREI (**Table 2**).

**Table 1:** ANOVA of GBEI, GLEI and GREI on genotypes and environments, and their interactions.

	GBEI		GLEI		GREI	
	F value	P value	F value	P value	F value	P value
Genotype (G)	11.279	2.5E-239	15.489	1.01E-306	48.364	0
Environment (E)	74.890	1.36E-17	1.843	0.1748481	130.473	6.15E-29
G×E	6.437	1.4E-138	7.070	1.35E-153	14.398	1.3E-290
Test of HOV	0.780	1.000	0.811	0.999	0.674	1.000

Test of HOV (homogeneity of variance) was performed using the method of Brown-Forsythe Test, in which *F*_0.05_ = 1.1134 (*df_1_* = 689, *df_2_* = 1380).

**Table 2:** Coefficients of correlation between different traits in each environment and between different environments in each trait.

	GBEI	GLEI	GREI
GBEI	0.317**	-0.101	-0.677**
GLEI	−0.155**	0.487**	0.750**
GREI	−0.767**	0.736**	0.542**

The data in the diagonal are correlations between the two years. The data in the lower triangle and the upper triangle are correlations between the three traits in E1 (2017) and in E2 (2018), respectively. ** indicates p-value < 0.01.

GREI exhibited significant positive and negative correlations with GLEI and GBEI, respectively (**Table 2**). This is understandable, as GREI is a composite trait that is influenced by both GLEI and GBEI. However, the correlation between GLEI and GBEI was found to be low (-0.155 in E1 and -0.101 in E2) (**Table 2**), implying that grain length expansion and breadth expansion during cooking are two relatively independent processes with potentially distinct genetic bases.

### 3.2 Population structures and linkage disequilibrium

**Figure 2:**
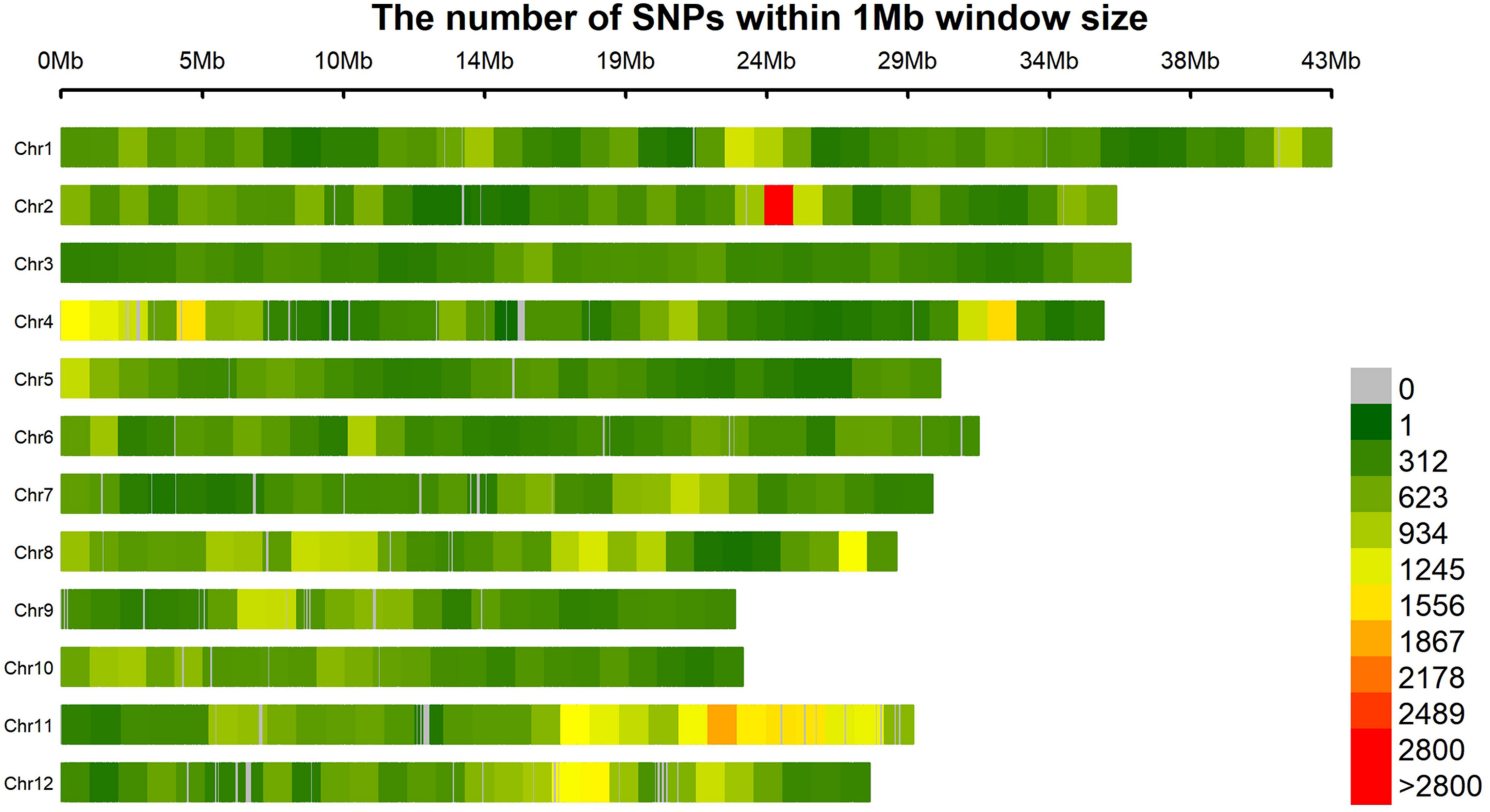
Distribution of 193,582 SNPs in the rice genome.

A set of 193,582 SNPs meeting the requirements of MAF > 5% and missing data < 20% were obtained. The SNPs were not evenly distributed in the genome (**Figure 2**). SNPs were the densest on chromosome 11 but the sparsest on chromosome 3, respectively (**Table 3**). On average, there was one SNP every 1928 bp in the genome.

**Table 3:** Number and density of SNPs and LD decay distances in the rice genome.

Chromosome	Number of SNPs	Average spacing (bp)	HDD (kb)	DD0.1 (kb)
1	20,083	2154.6	651.1	603.9
2	18,756	1916.0	158.4	62.1
3	13,674	2663.0	534.7	507.3
4	19,298	1839.7	223.1	94.6
5	12,058	2484.5	333.3	374.5
6	13,883	2250.9	419.5	420.3
7	13,389	2218.1	715.7	712.1
8	18,850	1508.9	513.7	295.8
9	10,978	2096.3	330.5	249.7
10	11,946	1942.7	688.1	388.2
11	24,068	1205.8	178.9	78.5
12	16,599	1658.6	485.2	83.9
Whole genome	193,582	1928.1	377.9	196.1

HDD, LD half-decay distance; DD0.1, distance of LD decay to 0.1.

**Figure 3:**
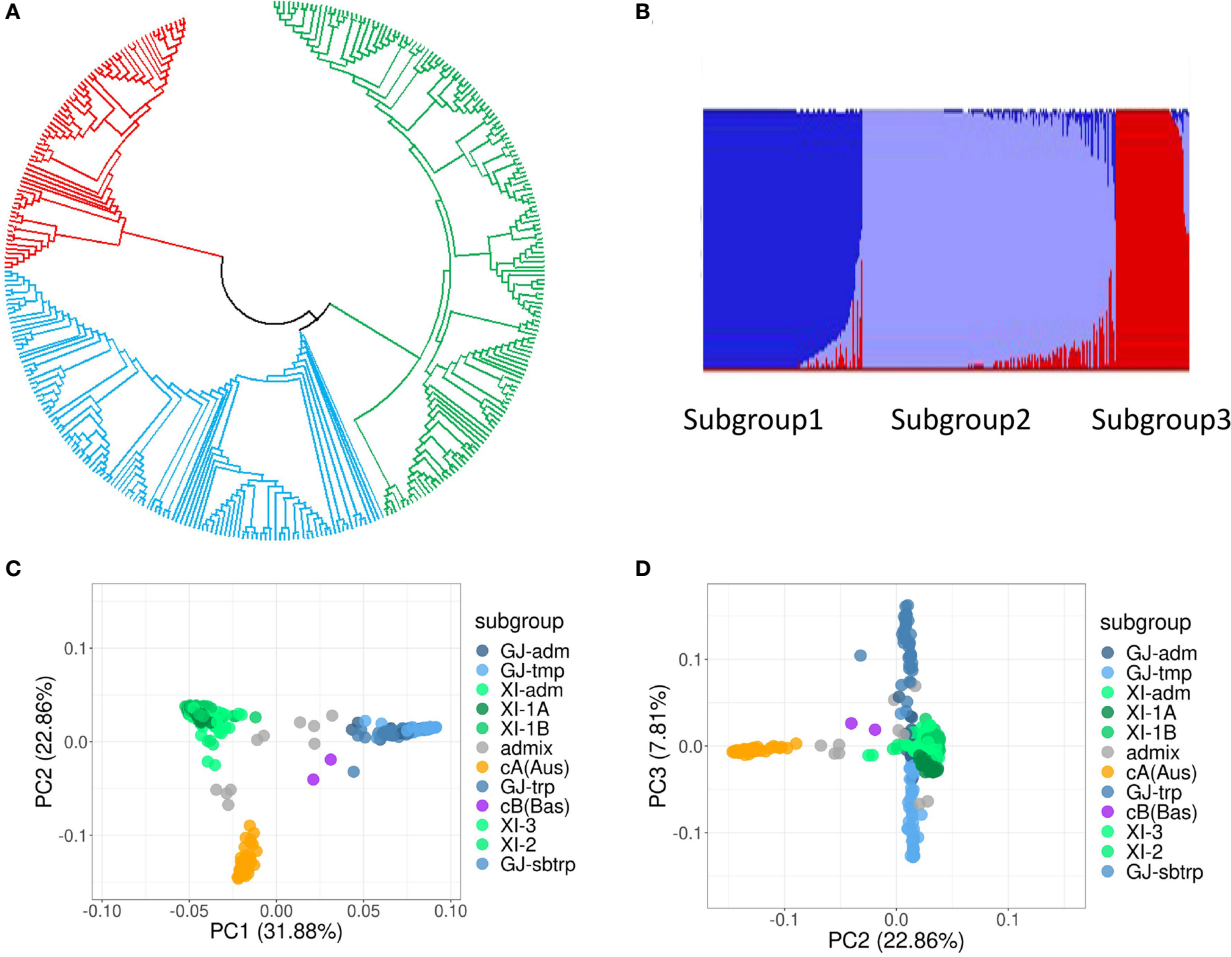
Genetic structure analysis of the population of 345 rice accessions. **(A)** Phylogenetic tree. **(B)** Population structure estimated by the software Admixture. **(C, D)** PCA plots of the first three principal components.

The results of phylogenetic analysis (**Figure 3A**), PCA (**Figure 3B**), and admixture analysis (**Figures 3C**, **D**) all indicated that the population of the 345 rice accessions could be basically divided into three distinct groups (subpopulations), namely, *indica* group, *japonica* group, and *aus* group (**Figures 3C**, **D**).

**Figure 4:**
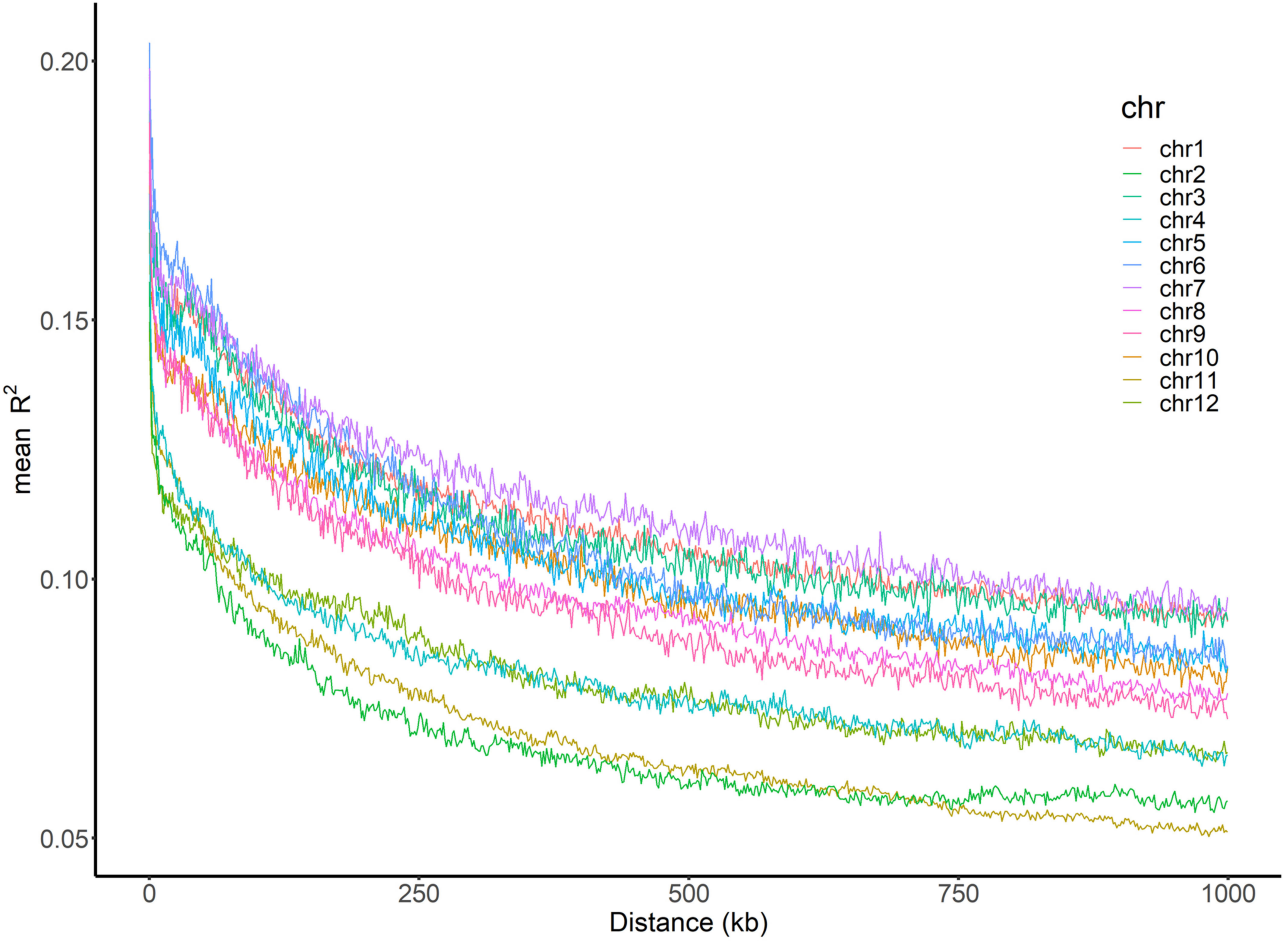
Genome-wide LD decay across 12 chromosomes. The x-axis represents the physical distance and the y-axis represents the average pairwise R^2^. The color of each chromosome was showed on the top right corner.

The average LD (mean R^2^) decreased with the increase of physical distance on every chromosome as well as in the whole genome (**Figure 4**). The average LD half-decay distance (HDD) and the average distance of LD decay to 0.1 (DD0.1) in the whole genome were about 378 kb and 196 kb, respectively (**Table 3**). However, the HDD and DD0.1 on different chromosomes varied greatly, ranging from 158.4 kb and 62.1 kb on chromosome 2 to 715.7 kb and 712.1 kb on chromosome 7, respectively (**Table 3**). Therefore, chromosome 2 had the highest LD decay rate, while chromosome 7 had the lowest.

### 3.3 QTNs detected by single-environment analysis

In total, 386 QTNs were detected by single-environment analysis using seven different methods, with 145, 127 and 128 QTNs found to be associated with GBEI, GLEI and GREI, respectively (**Table 4**; **Figures S1**, **S2**). However, only 78 (19.5%) QTNs were identified as reliable (**Tables 4**, **S2**). The total number of QTNs detected by each method varied greatly, ranging from 32 (FASTmrEMMA) to 131 (3VmrMLM; **Table 4**). The number and the percentage of reliable QTNs detected by each method also differed significantly (**Table 4**). Interestingly, there was a positive correlation between the number of reliable QTNs and the total number of QTNs detected by each method (correlation coefficient 80.5%), but a negative correlation between the percentage of reliable QTNs and the total number of QTNs detected by each method (correlation coefficient -88.2%). This indicates that the increase in the number of total QTNs and reliable QTNs detected by a method comes at the cost of a decrease in the percentage of reliable QTNs.

**Table 4:** Numbers of QTNs for GBEI, GLEI and GREI detected by seven methods in two different environments.

Method	GBEI			GLEI			GREI			Total^1^	Reliable QTNs
	E1	E2	Total^1^	E1	E2	Total^1^	E1	E2	Total^1^		
3VmrMLM	21	19	40	20	24	44	27	21	47	131	31 (23.5%)
mrMLM	10	9	19	7	6	13	16	7	23	55	25 (45.5%)
FASTmrMLM	13	49	62	11	19	29	12	10	22	114	40 (35.1%)
FASTmrEMMA	4	7	11	6	5	11	3	7	10	32	21 (65.6%)
pLARmEB	11	9	20	16	18	34	19	22	41	95	40 (42.1%)
pKWmEB	11	7	18	9	10	19	12	8	20	57	23 (40.4%)
ISIS EM-BLASSO	13	14	27	4	2	6	5	4	9	42	21 (50.0%)
Total^1^	56	90	145	55	73	127	70	60	128	400	78 (19.5%)

1. Redundancy was removed in the totals. 2. The number and proportion of reliable QTNs among the total detected by each method or in the whole experiment.

Among the three traits, GBEI had the most reliable QTNs, followed by GREI, and GLEI had the fewest (**Table 5**). Consistently, GBEI had highest proportion of phenotypic variance explained (PVE) by the reliable QTNs, followed by GREI, and GLEI had the lowest (**Table 5**). More reliable QTNs were detected and therefore there were higher PVEs in E1 than in E2 for GLEI and GREI, but the results in the two environments were similar for GBEI (**Table 5**).

**Table 5:** Statistics of reliable QTNs for GBEI, GLEI and GREI detected in each environment.

Trait	No. of reliable QTNs			LOD range		PVE range (%)		Total PVE (%)		
	E1	E2	Total	E1	E2	E1	E2	E1	E2	Average
GBEI	16	17	32	3.9-13.7	3.5-12.2	2.5-7.4	1.7-5.3	63.8	61.9	62.85
GLEI	13	6	19	3.2-14.3	3.9-8.5	0.2-7.2	0.1-4.0	41.5	12.8	27.15
GREI	16	12	27	3.6-14.9	3.9-12.9	1.3-7.7	0.0-9.0	47.8	36.8	42.30
Total	45	35	78							

PVE, proportion of phenotypic variance explained.

Most QTNs identified in this study were found to be reliable because they were detected by multiple methods, while only four QTNs (*qGBEI-5.4s*, *qGLEI-3.3s*, *qGREI-5.2s* and *qGREI-5.6s*) were found to be stable because they were detected in the two environments simultaneously (**Table S2**). In addition, there were a few SNPs exhibiting pleiotropic effects in one environment, including 3:16774870 (detected as QTNs *qGLEI-3.5s* and *qGREI-3.6s*) and 6:25062099 (*qGLEI-6.4s* and *qGREI-6.4s*), both of which were associated with GLEI and GREI; and 5:5369111 (*qGBEI-5.3s* and *qGREI-5.2s*), which was associated with GBEI and GREI (**Table S2**).

### 3.4 QTNs detected by two-environment analysis

**Figure 5:**
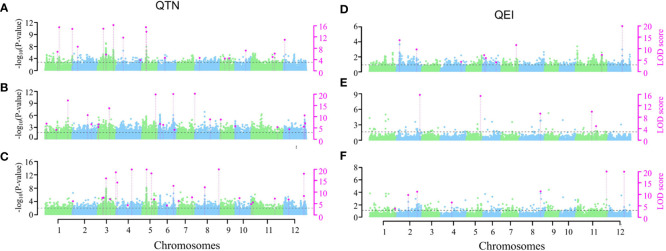
Manhattan plots of two-environment analyses on GBEI **(A, D)**, GLEI **(B, E)** and GREI **(C, F)**. The horizontal dashed lines indicate the LOD = 3.0 threshold. The left vertical axis is the -log_10_ (*P*-value), while the right vertical axis is the LOD score for each SNP marker. Pink dots indicate significant (-log_10_(*P*-value) ≥ 6.588) or suggested (-log_10_(*P*-value) < 6.588 but LOD ≥ 3) QTNs **(A–C)** or QEIs **(D–F)**.

The two-environment analysis detected 11, 14 and 19 significant QTNs (P-value ≤ 0.05/*m* = 2.58E-07, where *m* = 193,582, the number of markers) and 8, 8 and 6 suggested QTNs (P > 2.58E-07 but LOD ≥ 3.0) associated with GBEI, GLEI and GREI, respectively (**Figures 5A-C**; **Table S3**). These QTNs explained 35.41%, 46.37% and 41.49% of the total phenotypic variation in GBEI, GLEI and GREI, respectively. The SNP marker chr5:5369111 was found to be associated with both GBEI and GREI, and was named *qGBEI-5.2t* and *qGREI-5.3t*, respectively. This marker was also detected as QTNs *qGBEI-5.3s* and *qGREI-5.2s* in the single-environment analysis, indicating its reliability. Marker chr6:25000609 was associated with both GLEI and GREI, while chr11:23854971 was associated with both GBEI and GREI. Additionally, SNPs chr2:24264276, chr3:2521638, chr3:35669404 and chr5:14585838 were all detected in both single- and two-environment analyses.

The two-environment analysis also detected 6, 4 and 5 significant QEIs and 3, 1 and 2 suggested QEIs associated with GBEI, GLEI and GREI, respectively. These QEIs accounted for 24.83%, 14.79% and 21.22% of the total phenotypic variation in GBEI, GLEI and GREI, respectively (**Figures 5D-F**; **Table S4**). Notably, there was no common site between the QTNs and QEIs detected, indicating that all the SNPs exhibiting significant main (additive and/or dominance) effects in the two-environment analysis did not show significant effects of interaction with the environment, and vice versa (namely, all the SNPs exhibiting significant effects of interaction with the environment did not show significant main effects). Nonetheless, the SNP markers of two QEIs, *qGREI-2.3i* (SNP 2:19642336) and *qGLEI-5.6i* (SNP 5:25726382) were also detected as QTN *qGREI-2.2s* and *qGREI-5.8s* in the single-environment analysis, respectively (**Tables S2**, **S4**). Interestingly, the targeted traits of *qGLEI-5.6i* and *qGREI-5.8s* were not the same. In addition, the interaction between SNP marker 8:22185608 and environment was found to be associated with both GLEI (as *qGLEI-8.3i*) and GREI (as *qGREI-8.5i*) simultaneously (**Table S4**).

### 3.5 Prediction of candidate genes for GBEI, GLEI and GREI

**Figure 6:**
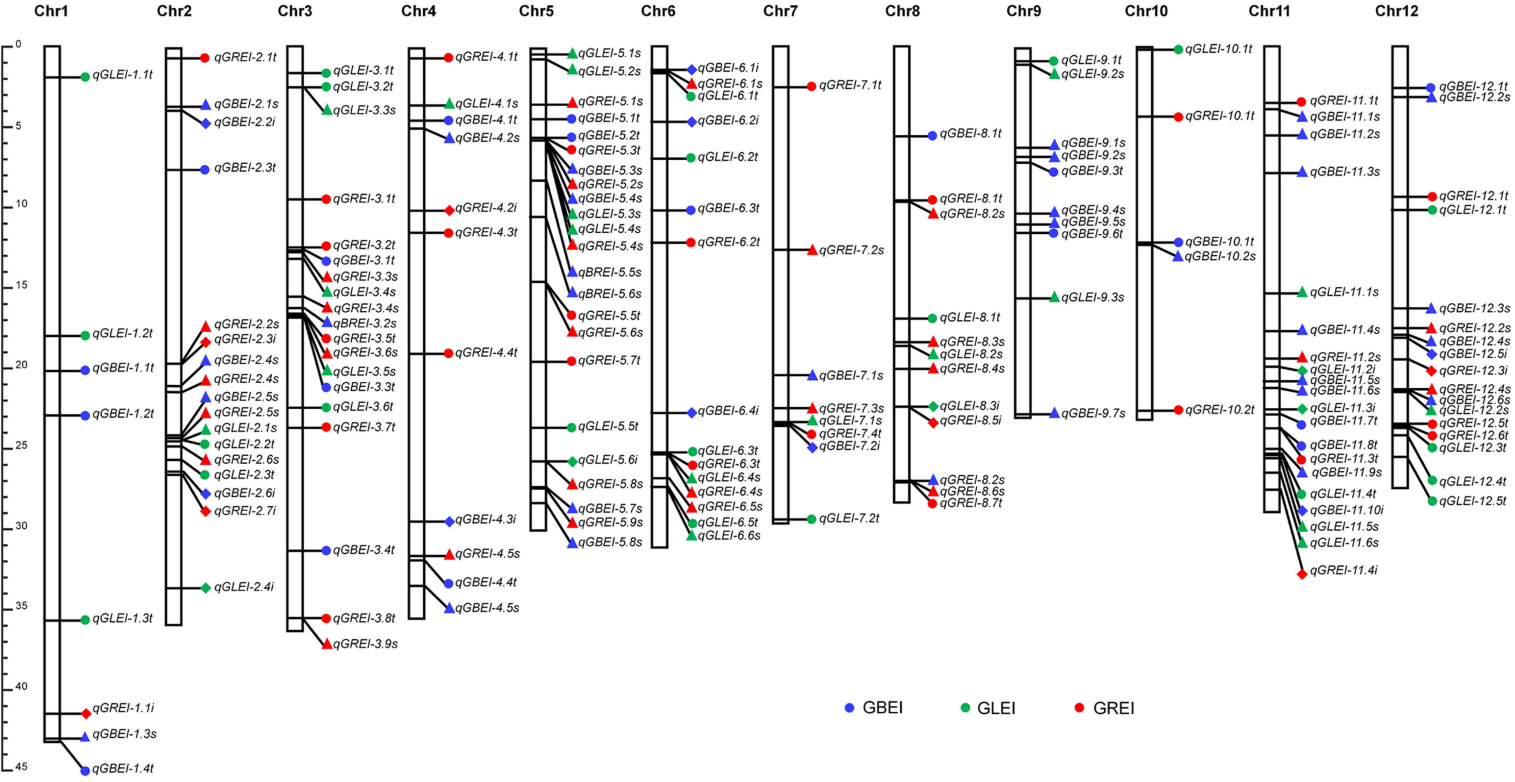
Locations of QTNs/QEIs for GBEI, GLEI and GREI in the rice genome. The QTNs detected by single-environment analysis are indicated by solid circles. The QTNs and QEIs detected by two-environment analysis are indicated by filled triangles and solid diamonds, respectively.

In total, the two-environment analysis detected 66 QTNs and 21 QEIs for the three traits. Plus the 78 reliable QTNs detected in the single-environment analysis, this study detected a total of 165 QTNs/QEIs. These QTNs/QEIs were mainly located on chromosomes 5, 11, 12, 3 and 2, and very rare on chromosomes 1 and 10 (**Figure 6**).

Considering that CCRGE may be largely determined by the starch in endosperm, we tried to predict the candidate genes involved in starch metabolism and endosperm development. By searching 20 related Gene Ontology/Term Ontology (GO/TO) classifications on the China Rice Data Center’s website (https://www.ricedata.cn/ontology/), 119 genes were found, of which 26 were located within the R^2^ half-decay distance around the detected QTNs/QEIs (**Table S5**). By analyzing the SNP variations in the genes with the software snpEff v1.9, five genes were found to carry effective mutations, including non-synonymous, splice site and UTR-5’ mutations (**Table 6**; **Figure S3**). So, these genes were considered to be candidate genes.

**Table 6:** Candidate genes for GBEI, GLEI and GREI .

Gene ID^1^	Gene name	Nearby QTN/QEI	Chr.	No. of Haplotypes	Mutation type	Annotation
*Os04g53310*	*OsSSIIIb*	*qGREI-4.5s*	4	5	non-synonymous, UTR-5’ mutation	soluble starch synthase 3, chloroplast precursor
*Os05g02070*	*OsMT2b*	*qGLEI-5.1s*	5	2	UTR-5’ mutation	metallothionein
*Os06g04200*	*wx*; *qGC-6*; *Wx-mq*; *Wx-op*	*qGBEI-6.1i*, *qGREI-6.1s, qGLEI-6.1t*	6	4	non-synonymous, UTR-5’ mutation	granule-bound starch synthase
*Os06g12450*	*ALK*; *OsSSIIa*	*qGLEI-6.2t*	6	3	non-synonymous, splice site mutation	soluble starch synthase 2-3, chloroplast precursor
*Os08g09230*	*OsSSIIIa*; *Flo5*	*qGBEI-8.1t*	8	2	non-synonymous mutation	starch synthase III

1. The full gene ID includes a prefix LOC_Os.

**Figure 7:**
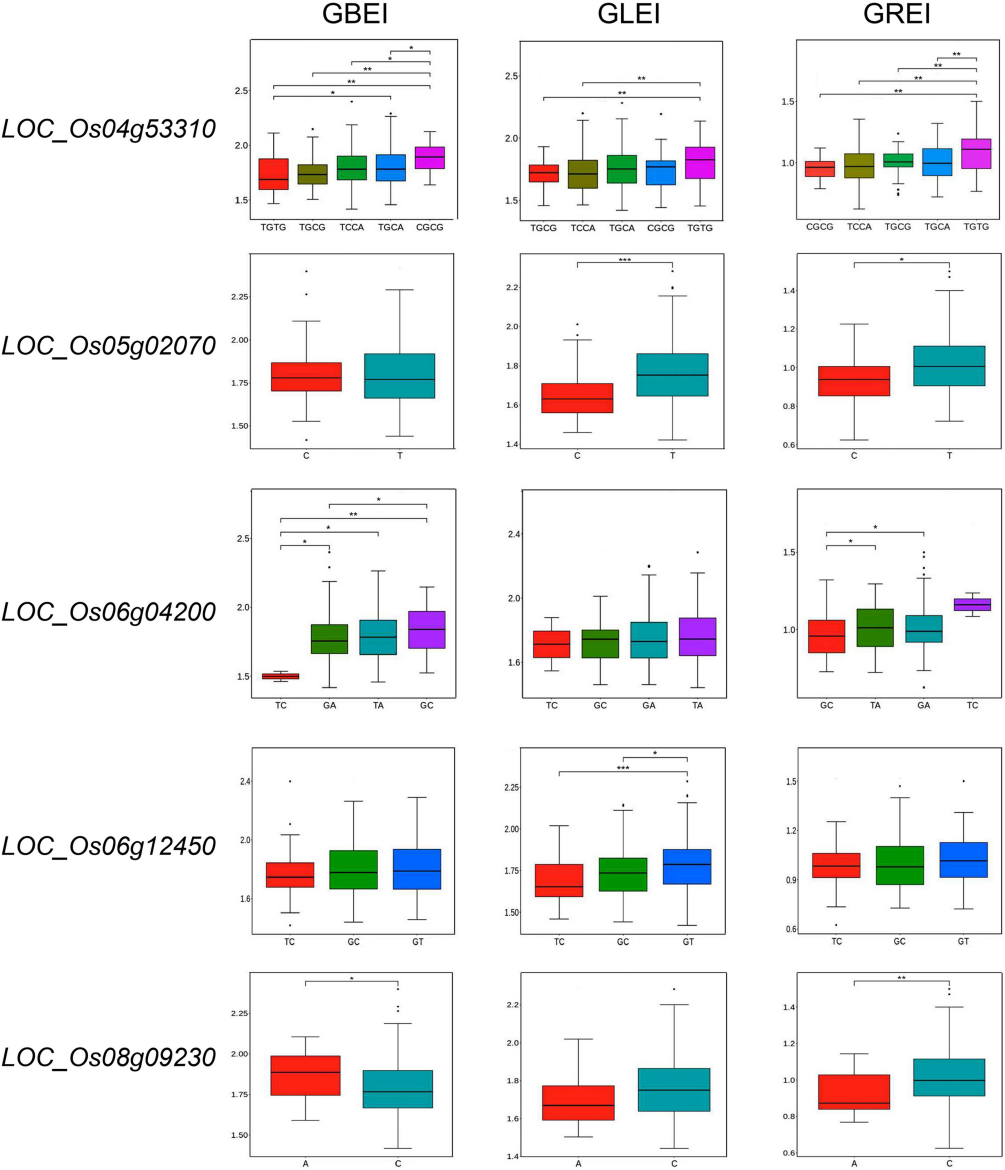
Haplotype analysis of candidate genes for GBEI, GLEI and GREI. *, ** and *** indicate significance at P<0.05, P<0.01 and P<0.001, respectively.

We then performed haplotype analysis to assess the reliability of the candidate genes. *LOC_Os04g53310* (*OsSSIIIb*), *LOC_Os06g04200* (*wx*) and *LOC_Os08g09230* (*OsSSIIIa*) exhibited significant haplotype differences for GBEI; *LOC_Os04g53310*, *LOC_Os05g02070* (*OsMT2b*) and *LOC_Os06g12450* (*OsSSIIa*) displayed significant haplotype differences for GLEI; and all of the genes except for *LOC_Os06g12450* showed significant haplotype differences for GREI (**Figure 7**). These findings strongly suggested a close association of these five genes with the CCRGE.

**Figure 8:**
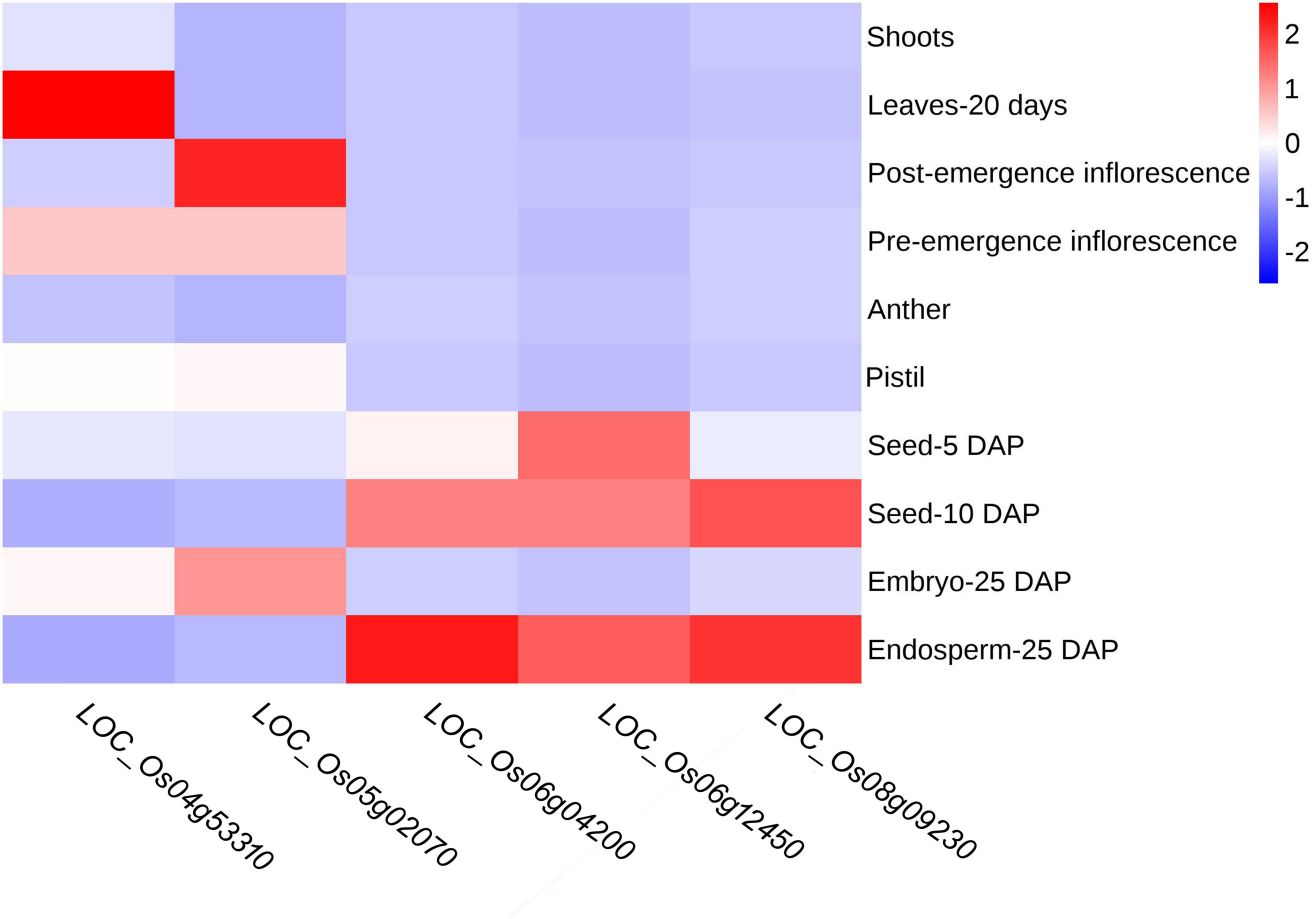
Heatmap of candidate gene expression analysis by RNA-seq data from RGAP database. Red boxes indicate high transcript levels and blue boxes indicate low transcript levels. DAP, days after pollination.

To further verify the potential impact of these candidate genes on the regulation of starch synthesis and endosperm development, we analyzed the expression patterns of the five candidate genes in various tissues based on data from the Rice Genome Annotation Project database (**Figure 8**). The results showed that *LOC_Os04g53310* (*OsSSIIIb*) was expressed mainly in leaf and pre-emergence inflorescence but not in seed or endosperm; *LOC_Os05g02070* (*OsMT2b*) was expressed mainly in post- and pre-emergence inflorescence and in embryo of 25 DAP (days after pollination), but not in endosperm. This suggests that these two genes maybe not closely or indirectly associated with endosperm development. In contrast, *LOC_Os06g04200* (*wx*), *LOC_Os06g12450* (*OsSSIIa*) and *LOC_Os08g09230* (*OsSSIIIa*) exhibited high expression in 10 DAP seed, and the highest expression in 25 DAP endosperm, but no expression in embryo, indicating their potential involvement in starch synthesis or endosperm development.

## 4 Discussion

When analyzing single environmental data, only QTNs, *qGREI-5.2s* and *qGREI-5.6s*, were commonly detected in two environments. This may be due to changes in the relative effects of different genes for these traits in different environments, indicating that the genes controlling these traits interacted with the environments. Joint analysis of the two environmental datasets using the 3VmrMLM method revealed 21 QEIs for three traits, also indicating the interaction between QTN and environment. Actually, ANOVA results showed significant genotype-by-environment interaction in the three traits (**Table 1**). However, there were no overlapping sites between QEI and QTNs detected based on two environmental data, indicating that all QEIs had no significant additive or dominant effect, but only the interaction effect between additive or dominant and environment, while all the QTNs in two-environment jointly analyze were opposite. Using the same 3VmrMLM method in previous studies, the overlapping sites between QEI and QTNs were also few, ranging from 1-3 sites (Han et al., 2022; He et al., 2022; Yu et al., 2022; Zhang et al., 2022; Jiang et al., 2023; Zhao et al., 2023), except for the study of Zou et al. (2022), which found 13 overlapping sites. From the perspective of the effect of QEI, since most QEIs do not have a significant additive or dominant effect, their reliability needs to be further confirmed.

In this study, among the 78 QTNs detected by single-environment analysis, only four QTNs were detected in both environments simultaneously (**Table 4**; **Supplemental Table 2**), indicating that only a small proportion (~5%) of QTNs exhibited stable significant effects across the environments. Interestingly, these four stable QTNs appear to represent four different types in terms of the way of being detected (**Supplemental Table 2**). The first type is *qGREI-5.6s*, which was detected by the same method in both environments, and no other methods detected it in either environment. The second type is *qGBEI-5.4s*, which was detected by one method in one environment, but by another method in the other environment. The third type is *qGREI-5.2s*, which was detected by one method in one environment, but by multiple other methods in the other environment. The fourth type is *qGLEI-3.3s*, which was also detected by one method in one environment and by multiple methods in the other environment, but with one method being the same in the two environments. It is noticeable that three of the four stable QTNs were detected in two different environments due to the use of multiple methods. These findings highlight the advantages of employing multiple GWAS methods to analyze the data collected from diverse environmental conditions.

According to the definitions, GREI is a composite trait that comprises various levels of component traits, which exhibit correlation with grain length and grain breadth before cooking (L_0_ and B_0_) or after cooking (L_1_ and B_1_), and is directly proportional to GLEI while inversely proportional to GBEI. Evidently, genes governing GBEI and GLEI may also impact GREI in principle. In other words, the QTLs for GREI may exhibit pleiotropic effects on its component traits or correlated traits. In this study, we did identify 4 QTNs that simultaneously influence GREI and GLEI, and 2 QTNs that simultaneously affect GREI and GBEI (**Table 7**). This was consistent with the high correlation between GREI and GLEI and GBEI (**Table 2**). As expected, there were no QTNs pleiotropic on GLEI and GBEI, which is in line with the conclusion that GLEI and GBEI are independent traits and have different genetic bases. Moreover, 3 QTNs controlling GLEI and GREI respectively were detected simultaneously in single and two environments, demonstrating the stability of these QTNs.

**Table 7:** Common QTLs between GREI, GBEI and GREI, or between different analysis aspects.

Chr.	QTN name	Trait	QTN pos. (bp)
2	*qGREI-2.2s, qGREI-2.3i*	GREI	19,642,336
2	*qGLEI-2.2s, qGLEI-2.2t*	GLEI	24,264,276
3	*qGLEI-3.3s, qGLEI-3.2t*	GLEI	2,521,638
3	*qGLEI-3.5s, qGREI-3.5s*	GLEI	16,774,870
3	*qGREI-3.9s, qGREI-3.8t*	GREI	35,669,404
5	*qGBEI-5.3s, qGREI-5.2s, qGBEI-5.2t, qGREI-5.3t*	GBEI, GREI	5,369,111
5	*qGREI-5.6s, qGREI-5.5t*	GREI	14,585,838
5	*qGLEI-5.6i, qGREI-5.8s*	GLEI, GREI	25,726,382
6	*qGLEI-6.3t, qGREI-6.3t*	GLEI, GREI	25,000,609
6	*qGLEI-6.4s, qGREI-6.4s*	GLEI, GREI	25,062,099
8	*qGLEI-8.3i, qGREI-8.5i*	GLEI, GREI	22,185,608
11	*qGBEI-11.8t, qGREI-11.3t*	GBEI, GREI	23,854,971

As mentioned above in the introduction, there were 10, 47 and 15 reported QTLs controlling length, width and length-width expansion caused by cooking in rice grain. Upon comparing these QTLs with the QTNs mapped in this study, we observed that 12, 9, and 15 QTNs for GBEI, GLEI, and GREI detected in this study were located within the intervals of one or more previously reported QTLs (**Table S6**). These comparisons provide evidence for the reliability of the QTLs detected in this study. Notably, the four putative genes (*LOC_Os05g02070*, *LOC_Os06g04200*, *LOC_Os06g12450*, and *LOC_Os08g09230*) identified in this study were found to be in close proximity to four of the aforementioned QTLs).

Due to the swelling of starch granules during cooking, rice grain cooking-caused expansion traits, such as GBEI, GLEI and GREI, is expected to be influenced by starch-related traits which include two typical traits: chalkiness rate and amylose content. Chalkiness rate is a crucial parameter for assessing the visual quality of rice, as high chalkiness rate can lead to easy breakage of grains during processing, low amylose content, and poor eating quality. Thi et al. (2020) utilized a genetic population to map GREI and discovered a positive correlation between amylose content and GREI, with high AC content leading to increased GREI. *OsMT2b* encodes a metallothionein that binds to metal ions and scavenges reactive oxygen species (ROS). Wu et al. (2022) reported that WCR1, a negative regulator of rice chalkiness rate, functions to regulate *OsMT2b* (*LOC_Os05g02070*) transcription level and inhibit 26S proteasome-mediated *OsMT2b* protein degradation, thereby facilitating ROS clearance, delaying programmed cell death (PCD) of endosperm cells, and ultimately increasing the accumulation of storage substances, and reducing chalkiness rate. In this study, a SNP site is present in the 5’-UTR region of *OsMT2b* near *qGLEI-5.1s* (**Figure S3**), which may disrupt the expression of *OsMT2b*, thereby affecting the change in rice cooking caused expansion in the analyzed population. Furthermore, considering the expression pattern of *OsMT2b*, it is noteworthy that its expression level exhibits a significant reduction in the endosperm. This observation implies its potential indirect influence on starch synthesis or endosperm development.

*wx* (*LOC_Os06g04200*), *OsSSIIa* (*LOC_Os06g12450*), and *OsSSIIIa* (*LOC_Os08g09230*) are crucial genes involved in the biosynthesis of starch in rice grains. *wx* gene encodes granule-bound starch synthase (GBSS), a major enzyme responsible for amylose synthesis (Kharshiing and Chrungoo, 2021). It exerts a direct influence on the amylose content in the endosperm and pollen of rice, as well as the gel consistency of grains (Su et al., 2011). *OsSSIIa* encodes a soluble starch synthase II, and mutations in this gene may affect the activity of starch synthase, which in turn affects the synthesis of medium-length branched chains of amylopectin, changes the crystal layer structure, and ultimately alters the gelatinization temperature (Gao et al., 2003). *OsSSIIIa* encodes soluble starch synthase III, the second key enzyme involved in rice starch synthesis (Zhou et al., 2016). Mutations in *OsSSIIIa* can affect the structure of amylopectin, amylose content, and physicochemical properties of starch in rice grains. Double mutants of *OsSSIIa* and *OsSSIIIa* exhibited increased chalkiness and amylose content, increased gelatinization temperature, and decreased viscosity (Zhang et al., 2011). In this study, these three genes exhibited the SNP loci with genetic effects. In haplotype analysis, significant differences in GBEI, GLEI, or GREI were observed across different haplotypes caused by SNPs within these genes. In expression pattern analysis, these three genes were highly expressed in the endosperm and seeds 10 days after pollination. All the evidence supported the hypothesis that these three genes were candidate genes controlling CCRGE.

In addition, *OsSSIIIb* (*LOC_Os04g53310*) is a gene that encodes soluble starch synthase in rice. Its expression level and activity directly impact the synthesis and quality of starch in rice endosperm. *OsSSIIIb* can interact coordinately with *OsSSIIIa*, and loss of function of both genes leads to an increase in resistant starch content in cooked rice (Wang et al., 2023). Although its protein function is redundant with OsSSIIIa, its expression pattern differs significantly from *OsSSIIIa* which is expressed in the endosperm. *OsSSIIIb* is mainly expressed in leaves but not endosperm (**Figure 8**). In this study, the five haplotypes generated by the four SNP loci contained in the *OsSSIIIb* gene exhibit significant differences in three traits. The evidence proves that *OsSSIIIb* may indirectly participate in starch sythesis and subsequently affect CCRGE.

## 5 Conclusion

In this study, data of GBEI, GLEI and GREI, three traits related to rice grain cooked expansion, were collected from 345 rice accessions in two distinct environments. Utilizing 193,582 SNP markers, seven methods were employed to identify QTNs based on single-environment data, while the 3VmrMLM method was utilized to identify QTNs and QEIs based on two-environment data. A total of 165 reliable QTNs/QEIs were detected, with 60, 46 and 59 of them being associated with GLEI, GBEI and GREI, respectively. Additionally, 26 genes related to starch synthesis or endosperm development were found to be located around these QTNs/QEIs. Further haplotype and expression pattern analyses led to the identification of five candidate genes, namely *LOC_Os04g53310* (*OsSSIIIb*), *LOC_Os05g02070* (*OsMT2b*), *LOC_Os06g04200* (*wx*), *LOC_Os06g12450* (*OsSSIIa*), and *LOC_Os08g09230* (*OsSSIIIa*). These findings can be instrumental in identifying genes and conducting in-depth genetic research on CCRGE.

## Data availability statement

The original contributions presented in the study are included in the article/**Supplementary Material**. Further inquiries can be directed to the corresponding author.

## Author contributions

YZ and WW conceived and designed the experiment. KT, LL, EK, EN, ML, WN and SA measured the phenotypes of the traits. YZ, KT and XX analyzed the data. YZ and KT wrote the draft. WW revised the manuscript. All authors contributed to the article and approved the submitted version.

## Supplementary material

Supplementary Figure 1 SNP site and its resulting mutation type in five candidate genes. The blue boxes represent exons; the horizontal purple lines represent introns; the white boxes represent 5’ or 3’-UTR. The direction of a white box indicates the direction of the gene in the genome.

Supplementary Figure 2 Manhattan plots of single environment anlalyses by six methods in mrMLM R package on GBEI **(A, D)**, GLEI **(B, E)** and GREI **(C, F)**. The horizontal dashed lines indicate the LOD = 3.0 threshold. The left vertical axis is the -log_10_ (*P*-value), while the right vertical axis is the LOD score for each SNP marker. Pink dots indicate QTNs detected by more than one method. Blue dots indicate QTNs detected by only one method.

Supplementary Figure 3 Manhattan plots of two-environment analyses by 3VmrMLM on GBEI **(A, D)**, GLEI **(B, E)** and GREI **(C, F)**. The horizontal dashed lines indicate the LOD = 3.0 threshold. The left vertical axis is the -log_10_ (*P*-value), while the right vertical axis is the LOD score for each SNP marker. Pink dots indicate significant (-log_10_(*P*-value) ≥ 6.588) or suggested (-log_10_(*P*-value) < 6.588 but LOD ≥ 3).
