# Supplementary figures and images for: Genome-wide association study of cooking-caused grain expansion in rice (Oryza sativa L.)
Source: Front Plant Sci. 2023 Aug 30;14:1250854. doi: 10.3389/fpls.2023.1250854 (PMC10498926; doi:10.3389/fpls.2023.1250854)

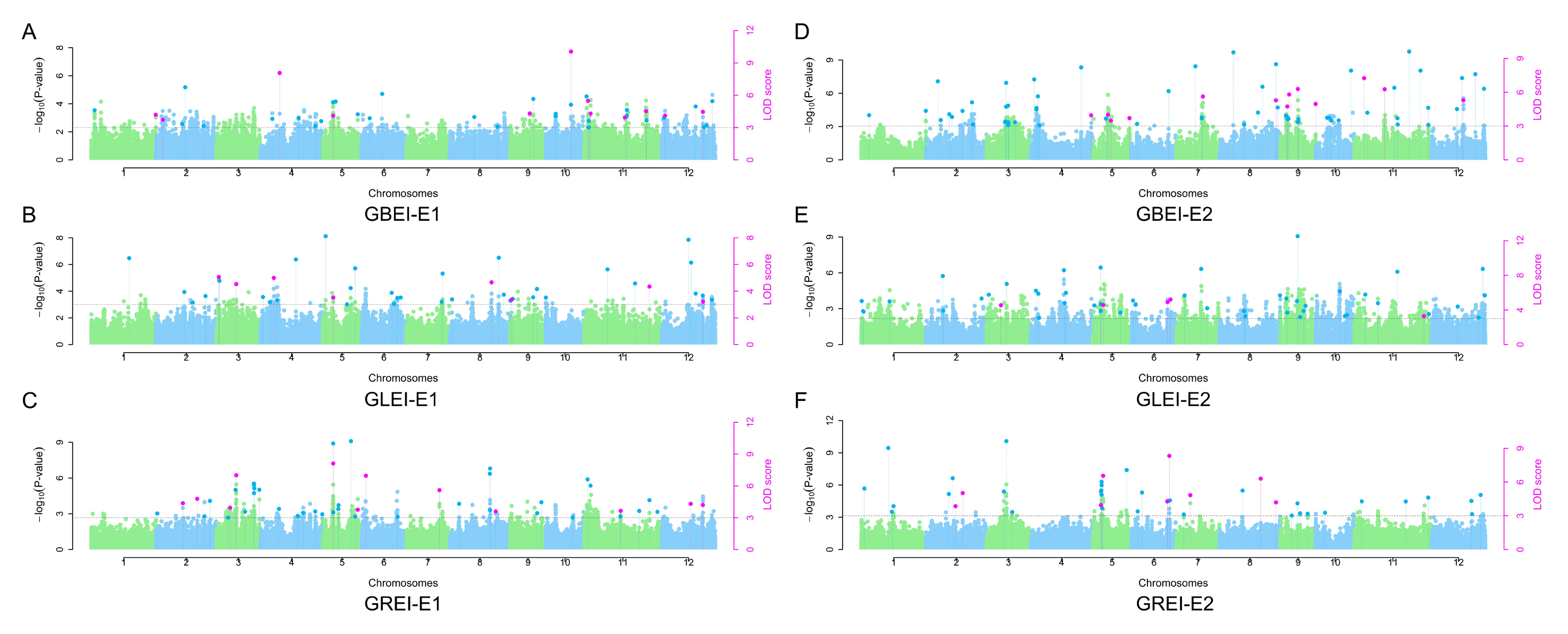

Supplement: Supplementary Figure 1 — SNP site and its resulting mutation type in five candidate genes. The blue boxes represent exons; the horizontal purple lines represent introns; the white boxes represent 5’ or 3’-UTR. The direction of a white box indicates the direction of the gene in the genome. [file Image_1.jpeg]

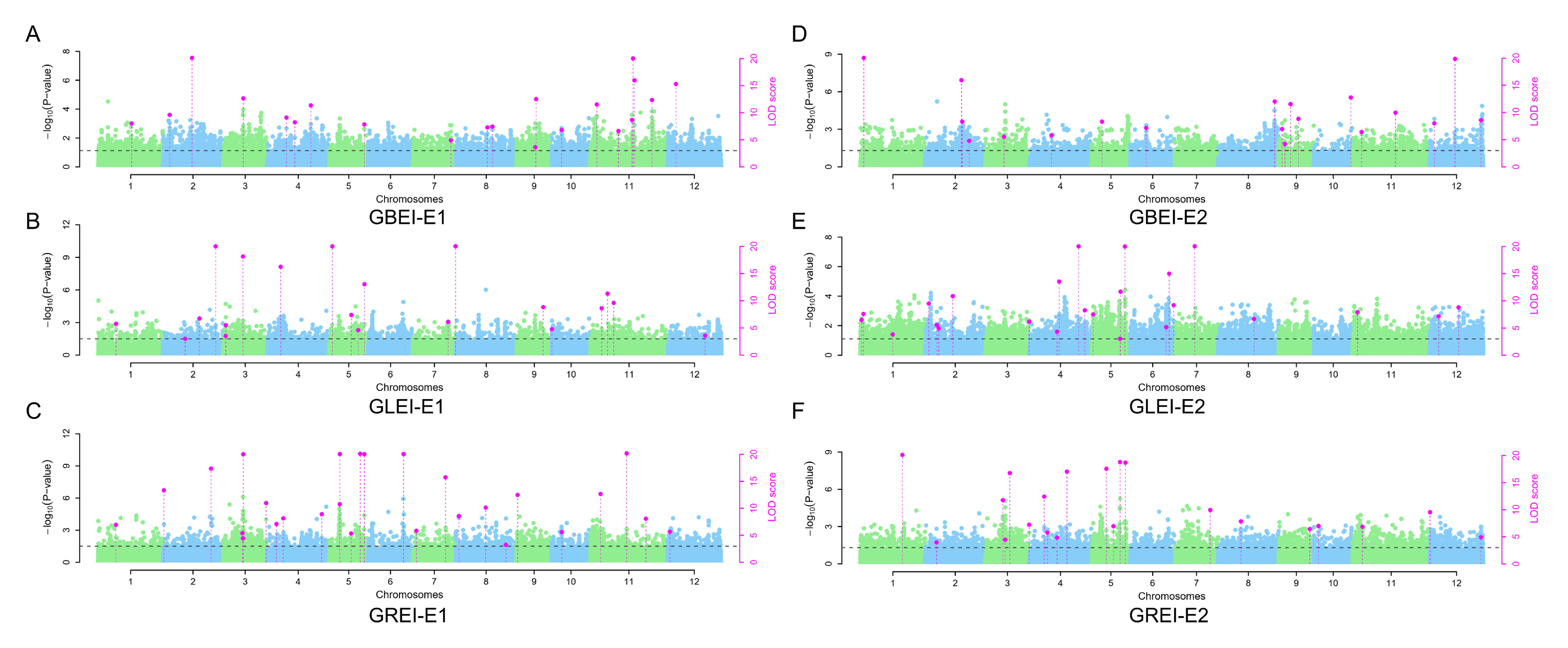

Supplement: Supplementary Figure 2 — Manhattan plots of single environment anlalyses by six methods in mrMLM R package on GBEI (A, D), GLEI (B, E) and GREI (C, F). The horizontal dashed lines indicate the LOD = 3.0 threshold. The left vertical axis is the -log10 (P-value), while the right vertical axis is the LOD score for each SNP marker. Pink dots indicate QTNs detected by more than one method. Blue dots indicate QTNs detected by only one method. [file Image_2.jpeg]

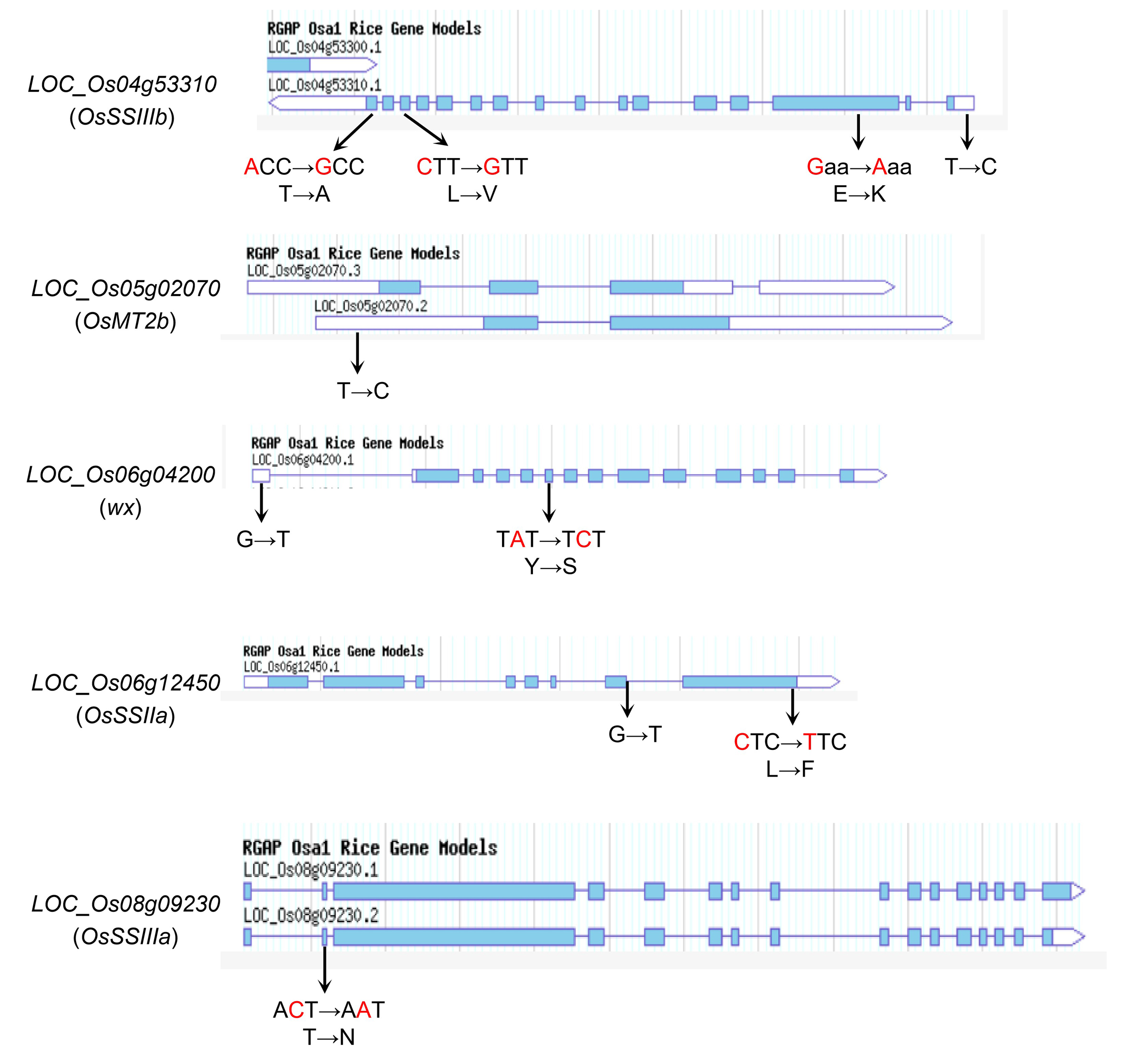

Supplement: Supplementary Figure 3 — Manhattan plots of two-environment analyses by 3VmrMLM on GBEI (A, D), GLEI (B, E) and GREI (C, F). The horizontal dashed lines indicate the LOD = 3.0 threshold. The left vertical axis is the -log10 (P-value), while the right vertical axis is the LOD score for each SNP marker. Pink dots indicate significant (-log10(P-value) ≥ 6.588) or suggested (-log10(P-value) < 6.588 but LOD ≥ 3). [file Image_3.jpeg]
